# A Dual Role of DDX3X in dsRNA-Derived Innate Immune Signaling

**DOI:** 10.3389/fmolb.2022.912727

**Published:** 2022-07-06

**Authors:** Juntae Kwon, Hyeongjwa Choi, Cecil Han

**Affiliations:** ^1^ Department of Oncology, Georgetown University School of Medicine, Washington, DC, United States; ^2^ Department of Biomedical Science and Technology, Konkuk University, Seoul, South Korea; ^3^ Lombardi Comprehensive Cancer Center, Washington, DC, United States

**Keywords:** innate immunity, cancer, type I IFN, DDX3X, dsRNAs

## Abstract

DEAD-Box Helicase 3 X-Linked (DDX3X) is essential for RNA metabolism and participates in various cellular processes involving RNA. DDX3X has been implicated in cancer growth and metastasis. DDX3X is involved in antiviral responses for viral RNAs and contributes to pro- or anti-microbial responses. A better understanding of how human cells regulate innate immune response against the viral “non-self” double-stranded RNAs (dsRNAs) and endogenous viral-like “self” dsRNAs is critical to understanding innate immune sensing, anti-microbial immunity, inflammation, immune cell homeostasis, and developing novel therapeutics for infectious, immune-mediated diseases, and cancer. DDX3X has known for activating the viral dsRNA-sensing pathway and innate immunity. However, accumulating research reveals a more complex role of DDX3X in regulating dsRNA-mediated signaling in cells. Here, we discuss the role of DDX3X in viral dsRNA- or endogenous dsRNA-mediated immune signaling pathways.

## Introduction

DDX3X is a member of the DEAD-box RNA helicase superfamily 2 (Linder and Jankowsky, 2011; Bourgeois et al., 2016). DEAD-box RNA helicases have ATP hydrolysis and RNA helicase activities to unwind RNA duplexes and participate in multiple aspects of RNA metabolism, such as transcription, RNA splicing, RNA transport, and initiation of the translation (Linder and Fuller-Pace, 2013; Ariumi, 2014; Shih and Lee, 2014; Song and Ji, 2019). DEAD-box RNA helicase consists of an N-terminal domain, two RecA-like domains that constitute the helicase core, and a C-terminal domain. The RecA-like domains contain 12 conserved motifs involved in ATP binding, RNA binding, and linking ATP hydrolysis with the RNA unwinding (Jarmoskaite and Russell, 2011; Linder and Fuller-Pace, 2013; Putnam and Jankowsky, 2013).

DDX3X is a multifunctional protein involved in cellular processes related to various human diseases. DDX3X mutations are linked to unexplained intellectual disability in female children (Johnson-Kerner et al., 1993; Snijders Blok et al., 2015). DDX3X has been linked to antiviral response against pathogenic invasion in mammals (Schröder et al., 2008; Soulat et al., 2008; Oshiumi et al., 2010; Gu et al., 2013; Liu and Gack, 2020). However, several RNA viruses, including human immunodeficiency virus type 1 (HIV-1) and Hepatitis C virus, exploit DDX3X for their replication, suggesting that DDX3X could be a pro- or anti-viral factor (Fullam and Schröder, 2013; Li et al., 2013; Valiente-Echeverría et al., 2015; Gringhuis et al., 2017; Kukhanova et al., 2020). The various functions of DDX3X in human disease have been extensively reviewed elsewhere (Bol et al., 2015; Kukhanova et al., 2020; Hernández-Díaz et al., 2021). This review will describe the complex role of DDX3X in the dsRNA-sensing pathway and Type I Interferon (IFN) response.

### Role of DDX3X in Viral dsRNAs and Innate Antiviral Immunity

Viral infection within cells often results in the production of dsRNAs, which are considered a “danger signal” sensed by the pattern recognition receptors (PRRs), which are part of the innate immune system (Schlee and Hartmann, 2016). Retinoic acid-inducible gene I (RIG-I)-like receptors (RLRs) and Nod-like receptors (NLRs) function as intracellular PRRs and are involved in innate antiviral immunity (Cao, 2009; Schlee and Hartmann, 2016; Gong et al., 2020; Onomoto et al., 2021). Upon recognizing “non-self’’ dsRNAs, the cellular dsRNA sensors, such as a retinoic acid-inducible gene I (RIG-I), melanoma differentiation-associated gene 5 (MDA5), protein kinase R (PKR), activate the dsRNA-sensing pathway and trigger antiviral responses *via* type I IFN production followed by upregulation of type I IFN-stimulated genes (ISGs), which eventually leads to antiviral state establishment (Ivashkiv and Donlin, 2014; Uggenti et al., 2019). Canonical type I IFN signaling leads to the transcription of hundreds of IFN-stimulated genes (ISGs) through the activation of the Janus kinase signal transducer and activator of transcription (JAK/STAT) pathway (Ivashkiv and Donlin, 2014).

DDX3X positively participates in type I IFN induction and antiviral response in mammalian cells at multiple levels (Fullam and Schröder, 2013; Hernández-Díaz et al., 2021; Ullah et al., 2022). First, DDX3X induces IFN production by direct association with antiviral signaling pathways (Schröder et al., 2008; Soulat et al., 2008; Li et al., 2013; Gu et al., 2017). DDX3X physically interacts with IκB kinase *ε* (IKK*ε*) (Schröder et al., 2008), TANK Binding Kinase 1 (TBK1) (Soulat et al., 2008), TNF Receptor Associated Factor 3 (TRAF3) (Gu et al., 2017), and Interferon regulatory factor 3 (IRF3) (Gu et al., 2013), resulting in enhanced type I IFN production upon viral infection or synthetic dsRNAs treatment in mammalian cells. Activation of the RIG-I pathway triggers the binding of DDX3X to IKK*ε*, which leads to an enhanced IKKϵ activation (Schröder et al., 2008; Gu et al., 2013). IKKε-mediated phosphorylation of DDX3X enables recruitment of interferon regulatory factor 3 (IRF3) into the complex, resulting in enhanced activation of IRF3 by IKKε phosphorylation and subsequent IFN-*α*/*β* gene transcription (Gu et al., 2013). Second, upon synthetic dsRNA poly (I:C) or viral dsRNAs stimulation, DDX3X forms a complex with mitochondrial antiviral-signaling protein (MAVS) to potentiate type I IFN responses. DDX3X acts as a viral RNA sensor independent of RIG-I and MDA-5 through a direct association with adaptor protein MAVS, which localizes upstream of TBK1 and IKKε, and triggers the MAVS-dependent signaling leading to type-I IFN production (Oshiumi et al., 2010). Third, TBK1 phosphorylates DDX3X, which allows the translocation of DDX3X to the nucleus, where it binds to the type I IFN promoter, leading to transcriptional activation of IFN-*α*/*β* genes at a transcription level (Soulat et al., 2008). These findings demonstrate DDX3X as a positive regulator for IFN production through the direct association with antiviral signaling pathways (Schröder et al., 2008; Soulat et al., 2008; Li et al., 2013; Gu et al., 2017).

During HIV-1 infection, the dual role of DDX3X in utilizing both proviral and antiviral capacities provides insight into its complexity and the various roles DDX3X might play in establishing immunity (Stunnenberg et al., 2020). Gringhuis et al. have shown DDX3X as an HIV-1 sensor that bound abortive HIV-1 RNA after HIV-1 infection and induced DC maturation and type I interferon responses *via* the signaling adaptor MAVS. However, during infection of DCs, HIV-1 hijacks DC-specific intercellular adhesion molecule-3 grabbing nonintegrin (DC-SIGN) function to block MAVS signaling, thereby preventing type I IFN and subsequent induction of antiviral innate and adaptive immune responses (Gringhuis et al., 2017; Stunnenberg et al., 2018). Interestingly, abortive HIV-1 RNAs consist of an HIV-1-specific complex secondary RNA hairpin-like structure, but lacking a poly-A tail. This complex structure of abortive HIV-1 RNAs, together with the 5′ cap, is required for binding to DDX3X, while absence of the poly-A tail prevents engagement of DDX3X with the cellular translational machinery (Gringhuis et al., 2017; Stunnenberg et al., 2020). Recently, Rao et al. revealed that targeting DDX3X reduces the HIV-1 latent reservoir using different latency models, including primary CD4^+^ T cells from people living with HIV under suppressive antiretroviral therapy. The authors show that DDX3X small molecule inhibitor treatment induced HIV-1 RNA expression, resulting in phosphorylation of IRF3, type I IFN, and nuclear factor kappa B (NF-κB) activation, which selective apoptosis of HIV-1-infected T cells (Rao et al., 2021). Especially, DDX3X inhibition results in latency reversal through viral RNA synthesis, leading to upregulation of NF-κB target genes and IRF3, followed by induction of IFN-*β* expression and a pro-apoptotic state, thereby rendering viral RNA-expressing cells more susceptible to cell death during viral RNA expression (Rao et al., 2021). These new data support the notion that DDX3X is a promising pharmacological target to treat HIV-1 infection at multiple levels (Hernández-Díaz et al., 2021; Rao et al., 2021).

In contrast to many previous studies of DDX3X enhancement of IFN-I induction upon viral infection (Hernández-Díaz et al., 2021), Loureiro and Zorzetto-Fernandes et al. demonstrated a negative role of DDX3X in IFN production in the context of arenavirus infection (Loureiro et al., 2018). This interesting study shows that DDX3 suppressed IFN-I production upon arenavirus infection, contributing to a DDX3X pro-viral effect late after arenavirus infection. They also showed that early after infection, pro-viral role of DDX3X was IFN-I independent and was mediated by facilitation of viral RNA synthesis *via* DDX3X ATPase and Helicase activities (Loureiro et al., 2018).

### Immunostimulatory Endogenous dsRNAs

Recent research has demonstrated that vertebrate cells cannot completely avoid the occurrence of endogenous self-nucleic acid structures with immunostimulatory properties (Schlee and Hartmann, 2016). Dysregulation of various “self” endogenous dsRNAs activates cytosolic dsRNA-sensing pathways, subsequently triggering antiviral immune responses in vertebrate cells without pathogenic infection (Katayama et al., 2005; Portal et al., 2015; Linder and Hornung, 2018; Bhate et al., 2019; Reich and Bass, 2019; Riley and Tait, 2020; Chen and Hur, 2021). Genomic repetitive elements such as short interspersed nuclear elements (SINEs), Long interspersed elements (LINEs), Endogenous Retroviral elements (ERVs), and mitochondrion-derived dsRNAs are primary sources of endogenous dsRNAs in mammalian cells (Kim et al., 2019). In addition, various cellular regulatory processes such as changes to RNA modifications, defects of dsRNA editing, splicing inhibition, deregulation of circular RNAs, defects in RNA processing and degradation, dysregulation of RNA Pol III, genotoxic stress, and mitochondrion-derived dsRNAs could be source for the endogenous dsRNAs (Chen and Hur, 2021).

The excessive formation, accumulation, or mislocalization of immunostimulatory endogenous dsRNAs can come with a significant risk for cells by the cell’s defense system against viruses. Therefore, tight containment of endogenous dsRNA within mitochondria, efficient transcriptional repression of repetitive elements, and retention of spurious transcripts in the nucleus are important (Dhir et al., 2018; Kim et al., 2019; Werner et al., 2021). Self-nucleic acid modification and nuclease-mediated degradation also provide additional mechanisms to diminish uncontrolled immune activation (Schlee and Hartmann, 2016). In addition, inhibition of polynucleotide phosphorylase (PNPase), which breaks down mitochondrial dsRNA, leads to cytosolic dsRNA sensing-pathway activation and ultimately triggers an interferon-mediated innate immune response (Dhir et al., 2018). Adenosine deaminase 1 (ADAR1) edits adenosine (A) of the double-stranded regions to inosine (I) (known as A-to-I RNA editing), which results in disruption of dsRNA structures or retention of edited dsRNAs in nucleus (Eisenberg and Levanon, 2018; Reich and Bass, 2019).

Some malignant cells have elevated expression of various endogenous dsRNAs, including transposable repetitive RNA elements, ERVs, mitochondrial dsRNAs, and structural dsRNAs, due to the loss of suppressive epigenetic modifications in repeat regions, genomic instability, or oxidative stress-induced mitochondrial damage, which could increase dsRNA burden in the cancer cells (Portal et al., 2015; Ahmad et al., 2018; Linder and Hornung, 2018; Tokuyama et al., 2018; Hur, 2019; Lamers et al., 2019; Reich and Bass, 2019). Aberrant overexpression of ERVs has been shown to increase the formation of dsRNAs in cancer cells (Chiappinelli et al., 2015; Jones et al., 2019). For example, DNA methylation inhibitor treatment led to the overexpression of ERVs and IFN pathway activation in cancer cells, which also increased the response of tumors to immunotherapy in *in vivo* animal studies (Chiappinelli et al., 2015; Roulois et al., 2015; Stone et al., 2017; Topper et al., 2017; Cañadas et al., 2018; Sheng et al., 2018). Inhibition of ADAR1 function leads to high cellular dsRNA stress and activation of IFN responses, increasing tumor sensitivity to the immunotherapy (Chung et al., 2018; Bhate et al., 2019; Ishizuka et al., 2019; Liu et al., 2019). These data suggest that modulation of cytosolic dsRNA sensors and immunostimulatory endogenous dsRNAs can be leveraged for therapeutic purposes by effectively inducing acute inflammation to promote anticancer immunotherapy (Riley and Tait, 2020).

### Regulatory Role of DDX3X in Endogenous dsRNAs and Human Diseases

**FIGURE 1:**
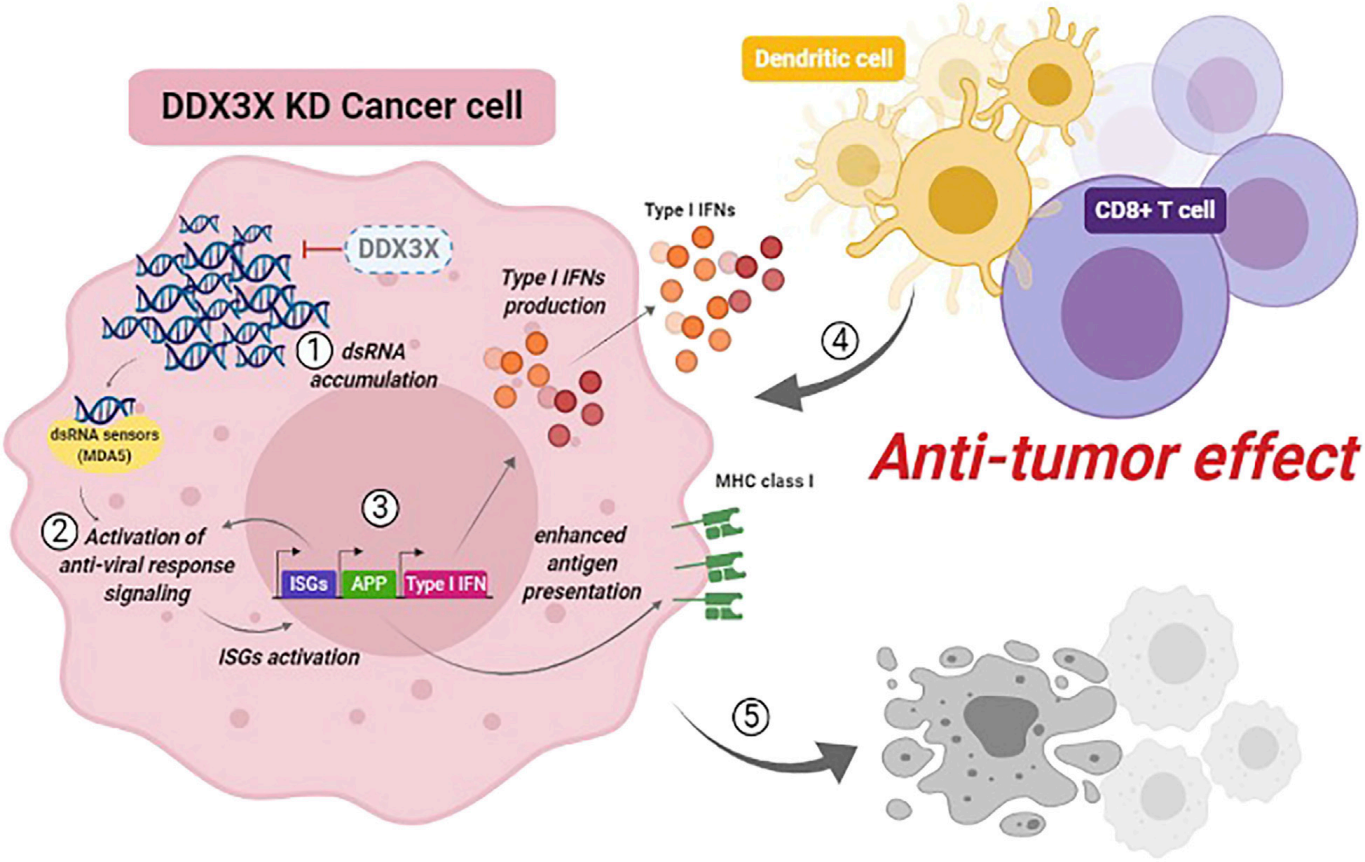
Increased endogenous dsRNA stress by targeting DDX3X in cancer. Inhibiting DDX3X leads to cytosolic accumulation of endogenous dsRNAs. Increased cellular endogenous dsRNA stress triggers cancer cell-intrinsic antiviral response *via* dsRNA-sensing pathway. Targeting DDX3X could enhance cancer cell-intrinsic innate immune response and boost anti-tumor immunity, suppressing tumor growth and metastasis.

Our study suggests that DDX3X prevents antiviral response against endogenous dsRNAs in the steady-state cell condition (without a viral infection or synthetic dsRNA treatment) (Choi et al., 2021). A small fraction of endogenous dsRNAs was detected in the cytoplasm. Still, these dsRNAs were not enough to induce an antiviral immune response in the homeostatic condition of the breast cancer cells. We found that 1) loss of DDX3X or inhibiting DDX3X resulted in the aberrant accumulation of endogenous dsRNAs in the cytoplasm of breast cancer cells; 2) the accumulation of dsRNAs activated the dsRNA-sensing pathway *via* mainly MDA5 and activation of nuclear factor kappa B (NF-κB) pathway followed by IFN-*β* production and enhanced antigen presentation in the DDX3X-depleted cells; 3) DDX3X-depleted cancer cells demonstrated the inhibited cancer proliferation and suppressed tumor growth in *in vivo* mouse studies. DDX3X-depleted breast tumor also exhibited tumor-intrinsic innate immune activation and increased tumor-infiltrated cytotoxic T cells and DCs in a syngeneic mouse model (Figure 1) (Choi et al., 2021).

It is still unclear whether or how DDX3X directly controls the levels, modification, or structure of endogenous dsRNAs that can activate the dsRNA-mediated immune response. Choi et al. showed that DDX3X interacts with two isoforms of ADAR1, suggesting DDX3X may provide an additional regulatory role in ADAR1-mediated dsRNA editing (Choi et al., 2021). Among two isoforms of ADAR1s, ADAR1-p110 (110 kDa) has been known to edit dsRNAs from Adenosine (A) to Inosine (I) mostly in the nucleus, while ADAR-1-p150 (150 kDa), functions in the cytoplasm. Interestingly, DDX3X primarily interacted with ADAR1-p150 in the cytoplasmic fraction of MCF7 cells (Choi et al., 2021). DDX3X knockdown alone increased the expression of IFN-beta and IFN-stimulated genes, comparable to the effect of ADAR1 knockdown in MCF7 breast cancer cells. Dual knockdown of DDX3X and ADAR1 produced an increased effect than single knockdown of each gene in upregulating the expression of type I IFN and IFN-stimulated genes. DDX3X depletion alone in MCF7 cells did not change the activity of ADAR1-mediated dsRNA editing, but the double knockdown of ADAR1 and DDX3X significantly reduced the dsRNA editing activity compared to ADAR1 depletion alone (Choi et al., 2021). These data suggest that double-depletion of DDX3X and ADAR1 may synergistically facilitate ADAR1-mediated dsRNA editing. These findings suggest that DDX3X could facilitate efficient dsRNA editing by unwinding complex three-dimensional RNA structures and exposing dsRNA extension to ADAR1.

DDX3X was co-precipitated with endogenous dsRNAs. DDX3X helicase activity was important in preventing the accumulation of immunostimulatory dsRNAs (Choi et al., 2021). Therefore, it is possible that DDX3X could act directly on dsRNAs by unwinding the dsRNA structures. Or DDX3X could be directly involved in the processing, degradation, or modification of endogenous dsRNAs. DDX3X has intrinsically disordered domains which may allow for DDX3X to guide DDX3X-associated dsRNAs to specific subcellular localizations *via* phase separation. DDX3X could bring other RNA-binding proteins to endogenous dsRNAs to control endogenous dsRNA homeostasis in cells. Identification of molecular machinery and characterization of dsRNAs regulated by DDX3X will be crucial to comprehending the complex role of DDX3X on endogenous dsRNAs.

Szappanos et al. showed that DDX3X could be essential not only for innate immunity against the infection of pathogens but also hematopoiesis and maintenance of immune cells. Mice lacking DDX3X in the hematopoietic system show alterations in the bone marrow and splenic cell populations and are highly susceptible to infection by pathogenic bacteria (Szappanos et al., 2018). *DDX3X* mutation is not common in most cancers. Still, recurrent mutations of *DDX3X* have been reported in medulloblastoma (a common childhood hindbrain tumor), mainly in wingless-type MMTV integration site family (WNT)-activated medulloblastomas and sonic hedgehog (SHH)-activated medulloblastomas (Jones et al., 2012; Northcott et al., 2012; Pugh et al., 2012; Robinson et al., 2012; Patmore et al., 2020). WNT medulloblastoma with *DDX3X* mutation revealed increased gene expression enriched in cell stress and immune response, implicating DDX3X role in inflammasome and innate immune signaling during medulloblastoma development mice models (Patmore et al., 2020). Recurrent mutations of *DDX3X* are also found in Natural killer (NK)/T-cell lymphoma (NKTCL), with a positive correlation between *DDX3X* mutations and poor clinical outcome (Jiang et al., 2015). Interestingly, *DDX3X* loss/mutation has revealed a different impact on proliferation of blood cancers. Compared to wild-type of DDX3X, overexpression of DDX3X mutants in NK cells increased cell proliferation with activation of NF-κB and MAPK pathways. Tumors with mutated *DDX3X* also exhibited upregulation of NF-κB and MAPK pathways. Notably, the mutations found in NKTCL are mostly related to the truncation or loss of DDX3X proteins, suggesting that DDX3X could function as a negative regulator of NK-cell proliferation through fine regulation of NF-κB and MAPK pathways (Jiang et al., 2015). Recurrent *DDX3X* mutations in diffuse large B-cell lymphoma (DLBCL) are associated with worse clinical outcomes. The mutation and loss of DDX3X in cell lines originated from DLBCL, cutaneous T-cell lymphoma (CTCL), and NK-cell/T-cell lymphoma (NKTCL) enhanced proliferation and STAT3/p42/p44 phosphorylation (Kizhakeyil et al., 2021). These studies suggest that DDX3X involve in development, differentiation, composition, and proliferation of immune cells through regulating innate immune signaling. The impact of expression and regulation of endogenous dsRNAs has not been studied in immune cells. Dysregulated endogenous dsRNAs induce type I IFN production, which may cause different biological impacts in immune cells and epithelial cells. Therefore, careful interpretation may be required to understand DDX3X mutations between blood and solid types of cancers.

Inflammasomes are innate immune system receptors and sensors that regulate the activation of caspase-1 and induce inflammation in response to infectious microbes and molecules derived from host proteins (Guo et al., 2015; Zhao and Zhao, 2020). Recent reports demonstrated that DDX3X is crucial for NOD-like receptor protein 3 (NLRP3) inflammasome assembly due to its direct binding interaction with NLRP3, and *Ddx3x* knockdown in peritoneal macrophages suppresses NLRP3 inflammasome activation and reduces pyroptosis (Samir et al., 2019). Samir et al. identified that DDX3X interacts with NLRP3 and is required for NLRP3 inflammasomes in bacterial lipopolysaccharides (LPS)-primed macrophages cells. Upon cells are stressed, pyroptosis mediated by NLRP3 inflammasome depends on availability of DDX3X, and DDX3X is also required for pro-survival assembly of stress granules (Samir et al., 2019). Kesavardhana et al. also showed that DDX3X-mediated NLRP3 activation in response to Influenza A virus infection (Kesavardhana et al., 2021). Kienes et al. demonstrate that NLRP11 can negatively regulate NLRP3 inflammasome activation *via* its interaction with DDX3X (Kienes et al., 2021). These studies show that DDX3X plays a molecular switch between cell pro-survival and pro-death responses by involving in nucleotide oligomerization domain (NOD)-like receptor protein 3 (NLRP3) inflammasome activation and stress granules assembly against cellular stresses (Samir et al., 2019; Kesavardhana et al., 2021; Kienes et al., 2021). Because cellular NLRP3 level is low enough to avoid aberrant inflammasome assembly and activation, NRLP3 inflammasome activation requires priming step, which is induced by PRRs activation upon virus infection. This leads to activation of NF-κB and promotes expression of NLRP3, pro-IL-1*β*, and pro-IL-18 (Guo et al., 2015; Zhao and Zhao, 2020). In our study, DDX3X depletion triggered NF-κB activation and type I IFN production in several breast cancer cells but did not promote the expression of NLRP3, pro-IL-1*β*, and pro-IL-18 (data not published). Sequestration of DDX3X into cytosolic stress granules during cellular stress resulted in reduced NLRP3/caspase-1 activation and suppression of IL-1*β* and IL-18 release, showing that NLRP3 inflammasome depends on availability of DDX3X (Samir et al., 2019). Type I IFNs was shown to reduce expression of pro-IL-1*β* and pro-IL-18 and repress NLRP3 inflammasome activity (Guarda et al., 2011). DDX3X may play a fine regulatory role between type I IFN-induced innate immunity and NLRP3 inflammasome-mediated innate immunity. NLRP3 inflammasomes can also be activated by dsRNAs (Zhao and Zhao, 2020). Given the conflicting role of DDX3X in inducing type I IFN production against viral dsRNAs and host dsRNAs, it will also be interesting to investigate whether DDX3X may function differently in inflammasome activation against endogenous cellular dsRNAs.

## Summary

**FIGURE 2:**
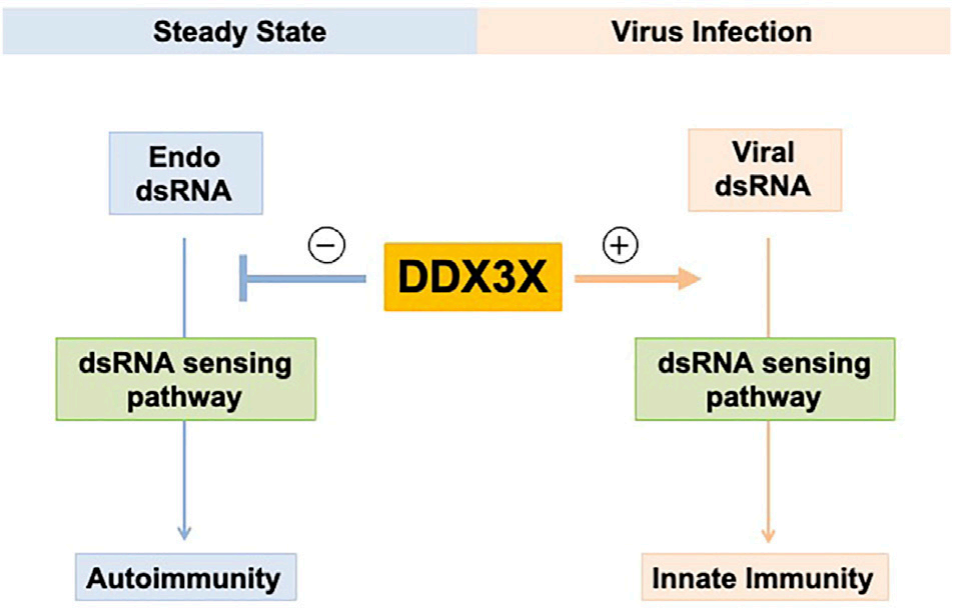
Potential dual role of DDX3X in regulating dsRNAs and innate immunity. DDX3X promotes innate antiviral immunity by activating the dsRNA-sensing pathway and type I IFN production upon viral infection and exogenous dsRNA stimulation. DDX3X is involved in endogenous cellular dsRNA homeostasis in the steady-state cells to prevent autoimmunity against endogenous dsRNAs.

Accumulating research has revealed a complex role of DDX3X in dsRNA biology network and antiviral immunity. Upon stimulation of viral dsRNAs or viral mimicking synthetic dsRNAs, DDX3X often promotes antiviral response *via* positively regulating type I IFN production. However, DDX3X seems to prevent autoimmunity against endogenous cellular dsRNAs in normal or malignant cells in steady-state condition, suggesting that DDX3X may have a “dual effect” on immune activity in humans (Figure 2). Human threatening viruses such as HIV-1 and Hepatitis C virus have evolved to exploit DDX3X’s functions for their viral replication. Understanding this exciting contradiction in DDX3X function will help us not only to improve our knowledge of virus-host interactions but also to develop novel antiviral drugs targeting the multifaceted roles of DDX3X in viral replication. DDX3X has been considered a promising anticancer target because of its predicted druggability and involvement in tumorigenesis (Fuller-Pace, 2013; Bol et al., 2015; Samal et al., 2015; van Voss et al., 2015; Xie et al., 2015; Heerma van Voss et al., 2018; Kukhanova et al., 2020). To better exploit DDX3X for cancer therapy, it will be important to investigate whether DDX3X-dsRNA-innate immune response axis would impact tumorigenesis and further determine if cancer-associated DDX3X mutations could be functionally related to this DDX3X-dsRNAs regulatory axis. Furthermore, it will be interesting to examine whether cancer cells control DDX3X level or activity to evade immune detection in tumor microenvironment. Inhibiting DDX3X may restore cancer immunity and enhance the antitumor activity by inducing dsRNA-derived type I IFN response in tumors, leading to novel agents targeting DDX3X for combinatory immunotherapy. In conclusion, understanding the role of DDX3X in the interplay between innate immune sensing and regulating endogenous dsRNAs has essential implications for understanding immune responses to human threatening viruses and tumors.
